# Factors associated with the local control of brain metastases: a systematic search and machine learning application

**DOI:** 10.1186/s12911-024-02579-z

**Published:** 2024-06-21

**Authors:** Hemalatha Kanakarajan, Wouter De Baene, Karin Gehring, Daniëlle B. P. Eekers, Patrick Hanssens, Margriet Sitskoorn

**Affiliations:** 1 Department of Cognitive Neuropsychology, Tilburg University, Tilburg, The Netherlands; 2 Gamma Knife Center, Elisabeth-TweeSteden Hospital, Tilburg, The Netherlands; 3 Department of Neurosurgery, Elisabeth-TweeSteden Hospital, Tilburg, The Netherlands; 4 Department of Radiation Oncology (Maastro), GROW School for Oncology and Reproduction, Maastricht University Medical Centre+, Maastricht, The Netherlands

**Keywords:** Local control, Brain metastases, SRT dose, Tumor volume, Local control factors

## Abstract

**Background:**

Enhancing Local Control (LC) of brain metastases is pivotal for improving overall survival, which makes the prediction of local treatment failure a crucial aspect of treatment planning. Understanding the factors that influence LC of brain metastases is imperative for optimizing treatment strategies and subsequently extending overall survival. Machine learning algorithms may help to identify factors that predict outcomes.

**Methods:**

This paper systematically reviews these factors associated with LC to select candidate predictor features for a practical application of predictive modeling. A systematic literature search was conducted to identify studies in which the LC of brain metastases is assessed for adult patients. EMBASE, PubMed, Web-of-Science, and the Cochrane Database were searched up to December 24, 2020. All studies investigating the LC of brain metastases as one of the endpoints were included, regardless of primary tumor type or treatment type. We first grouped studies based on primary tumor types resulting in lung, breast, and melanoma groups. Studies that did not focus on a specific primary cancer type were grouped based on treatment types resulting in surgery, SRT, and whole-brain radiotherapy groups. For each group, significant factors associated with LC were identified and discussed. As a second project, we assessed the practical importance of selected features in predicting LC after Stereotactic Radiotherapy (SRT) with a Random Forest machine learning model. Accuracy and Area Under the Curve (AUC) of the Random Forest model, trained with the list of factors that were found to be associated with LC for the SRT treatment group, were reported.

**Results:**

The systematic literature search identified 6270 unique records. After screening titles and abstracts, 410 full texts were considered, and ultimately 159 studies were included for review. Most of the studies focused on the LC of the brain metastases for a specific primary tumor type or after a specific treatment type. Higher SRT radiation dose was found to be associated with better LC in lung cancer, breast cancer, and melanoma groups. Also, a higher dose was associated with better LC in the SRT group, while higher tumor volume was associated with worse LC in this group. The Random Forest model predicted the LC of brain metastases with an accuracy of 80% and an AUC of 0.84.

**Conclusion:**

This paper thoroughly examines factors associated with LC in brain metastases and highlights the translational value of our findings for selecting variables to predict LC in a sample of patients who underwent SRT. The prediction model holds great promise for clinicians, offering a valuable tool to predict personalized treatment outcomes and foresee the impact of changes in treatment characteristics such as radiation dose.

**Supplementary Information:**

The online version contains supplementary material available at 10.1186/s12911-024-02579-z.

## Introduction

Brain metastases represent the most common intracranial tumor in adults [1]. An estimated 20% of all patients with cancer will develop brain metastases [2]. Although brain metastases can occur from any cancer, the three most common primary tumors associated with brain metastases are lung (20–56% of patients), breast (5–20%) and melanoma (7–16%) [3]. Advances in the treatment of primary tumors have led to prolonged life expectancy and therefore increased the probability of developing brain metastases [1]. Although some patients who develop brain metastases remain asymptomatic, many patients show neurological symptoms including headaches, nausea, vomiting, dizziness, focal neurological deficits, epileptic seizures, behavioral changes, and cognitive impairment [3, 4]. The overall prognosis for patients with brain metastases remains poor [5]. Brain metastases account for a disproportionately high percentage of morbidity and mortality among patients with cancer [6], with dismal 2- and 5-year survival rates of 8.1% and 2.4% respectively after diagnosis [3].

Conventional local treatment options for brain metastases include surgical resection, Whole Brain Radiotherapy (WBRT), Stereotactic Radiotherapy (SRT), or a combination of these. Surgery is a treatment option for large metastatic brain lesions [7]. With WBRT, the entire brain, including healthy brain tissue, is irradiated with a fractionated treatment regimen. WBRT used to be the standard of care for multiple brain metastases. Since long-term adverse cognitive decline is a common neurotoxic effect in patients who have undergone WBRT, and SRT has become increasingly available, SRT is currently generally performed to avoid these cognitive side effects of WBRT [8, 9]. Some studies found SRT to be an effective treatment option for patients with multiple brain metastases [10–14]. As per the joint practice guidelines from the European Association of Neuro-Oncology (EANO) and the European Society for Medical Oncology (ESMO), SRT is recommended for patients with a limited number (1–4) of brain metastases and SRT may be considered for patients with a higher number of brain metastases (5–10) with a cumulative tumor volume < 15 ml [191]. SRT to the surgical cavity is a reasonable option for patients with one to two resected brain metastases [15]. The clinical trial of Brown et al. [192] showed that postoperative SRT is a superior alternative to WBRT for patients with a single brain metastasis.

Irrespective of the treatment type, LC of brain metastases remains an important clinical endpoint [16]. LC is defined as the freedom from the development of new lesions within the field treated with SRT or the absence of progression in preexisting metastases [17, 18]. The prediction of the LC of brain metastases after treatment has important practical implications for patients and clinicians. A predictive capability of the treatment outcome of brain metastases may provide a decision tool to clinicians for the effective management of patient care with the most desirable treatment outcome. If LC can be predicted, the treatment plan can be modified to improve LC by, for example, increasing the dose [19]. The complexity of predicting LC post-treatment remains, however, a critical challenge.

Machine learning, which entails a set of tools and structures to acquire information from data [20], has emerged as a promising avenue for predicting treatment outcomes [21, 22]. Machine learning presents important advantages in predictive performance and in the ability to account for complex interactions among inputs while scaling to data sets of very large sizes [23]. These models have shown great success in disease risk predictions based on historical clinical data. Recently, several studies relied on machine learning techniques to predict the response of brain metastases to SRT with high accuracy. Kawahara et al. [19], for instance, proposed a neural network model for predicting the local response of metastatic brain tumors to SRT. The study of Jaberipour et al. [24] investigated the effectiveness of pre-treatment quantitative Magnetic Resonance Imaging (MRI) and clinical features with machine learning techniques to predict local control in patients with brain metastasis treated with SRT. Jalalifar et al. [187] introduced a novel deep learning architecture to predict the LC in brain metastasis treated with SRT using pre-treatment MRI and standard clinical attributes.

However, the complexity of machine learning models and their limited interpretability pose challenges, particularly in biomedical and clinical areas where interpretability is crucial [27]. The inclusion of redundant features for training a machine learning model for the prediction of the local control of brain metastases will also degrade the performance of the model and increase the computation time. Additionally, the inclusion of redundant and irrelevant features reduces the model’s ability to generalize to unseen datasets. Feature selection is a crucial step in minimizing the problem of excessive and irrelevant features and enhancing model interpretability [28]. However, currently, there is lack of insight into the factors influencing local control of brain metastases irrespective of primary tumor types and treatment types. A systematic review of the factors influencing local control may provide the clinical insights needed to select the relevant factors.

Recognizing the importance of understanding the predictors of LC, our paper systematically reviews factors associated with LC of brain metastases. Unlike previous reviews confined to specific primary tumor types or treatment modalities [25, 26], our approach aims to provide a holistic overview of factors associated with LC, encompassing all treatment types, all primary tumor types and all characteristics associated with LC. The comprehensive nature of this review provides the foundation for machine learning model development.

Our study leverages the findings from our comprehensive review to perform feature selection for a Random Forest machine learning algorithm to predict LC specifically for the brain metastases patients treated with SRT group. Experimental results comparing different approaches showed that the Random Forest machine learning algorithm has better prediction performance than logistic regression in approximately 69% of the 243 real datasets [29], including 77 biological datasets, and the experiment compared the prediction performance of the Random Forest algorithm with that of logistic regression for a wide range of prediction outcomes. Also, the Random Forest algorithm performed better than other classification algorithms like support vector machines, K-nearest neighbors, and linear discriminant analysis [30]. Hence, we chose the Random Forest machine learning algorithm to find the importance of the factors and to predict LC for the SRT treatment group.

Our paper strives to bridge the gap between clinical insights and machine learning applications by providing a comprehensive overview of candidate predictors for LC of brain metastases. We use the Random Forest model as an illustrative example, highlighting the potential integration of machine learning in understanding and predicting treatment outcomes. This approach underscores the importance of unraveling predictors to pave the way for future advancements in personalized and effective cancer care.

## Methods

### Literature search

We conducted this systematic literature review according to the Preferred Reporting Items for Systematic Reviews and Meta-Analysis (PRISMA) guidelines [190]. A systematic literature search was conducted to identify studies in which the LC of brain metastases was assessed for adult patients. EMBASE, PubMed, Web-of-Science, and the Cochrane Database were searched up to December 24, 2020. Inclusion criteria were studies investigating factors associated with LC of brain metastases. All studies investigating the LC of brain metastases were included irrespective of the primary tumor type. Also, there were no limitations based on the types of treatment.

Eligible studies were research papers, clinical studies, clinical trials, controlled trials, comparative studies, evaluation studies, journal articles, meta-analyses, and case series published since 2010, written in English. Systematic reviews and narrative reviews were also included in the search criteria.

Broad search terms were used to ensure that all studies investigating LC would be covered in the search. The studies were screened to select all those that included LC of brain metastases as an endpoint. Studies that did not have LC as an endpoint were excluded.

The inclusion and exclusion criteria in terms of PICOs (population, intervention, comparison, outcome) are presented in Supplementary Table 1 The search terms are presented in Supplementary Table 2 The inclusion, exclusion, and search terms were built by the first and second authors and reviewed by the other authors.

### Study selection

All studies were screened by the first (HK) and second author (WDB) based on title and abstract. The full text was screened if it was unclear from the abstract whether the study met the inclusion criteria. Screening results from both authors were compared and cases of doubt were discussed. Consensus was reached in all cases.

### Assessment of included studies

The important factors that were critically reviewed were the aim of the study, primary tumor type, primary and secondary endpoints, treatment type, and the factors associated with the LC of brain metastasis. From the papers that met the inclusion criteria, the significant factors associated with LC were noted. If both univariate and multivariate results were reported, all factors reaching significance in at least one of the two analyses were recorded. We also looked at non-significant univariate factors (neither significant in the univariate analysis nor the multivariate analysis) to report on contradictory findings about the association of a factor across different studies, as some significant factors in some studies might not be significant in others. If the outcome of the analysis was not clear (e.g., direction of the effect was not reported), the corresponding study was excluded.

### Presentation of results

We first grouped studies based on primary tumor types. The three groups we created based on the primary tumor types were lung, breast, and melanoma. Studies that did not focus on a specific primary tumor type but included heterogeneous groups of patients with diverse primary cancer types were grouped based on treatment type. The groups we created based on the brain metastases treatment type were a surgery, an SRT, and a WBRT group. The results section visually depicts the significant factors associated with better and worse LC in each group. The factors not associated with LC and the factors for which there are mixed findings are not included in these figures but are added as text in the results section.

In the results section, we combined all results in the same direction for continuous variables like radiation dose, tumor volume, tumor size, and age. For example, some papers mention that a higher dose is associated with better LC whereas others mention that a lower dose is associated with worse LC. In the visual depictions, we have included higher dose under the list of factors associated with better LC and added the reference of all these papers.

### Machine learning use case

We retrospectively collected the clinical data from 200 brain metastases patients from the Gamma Knife Center of the Elisabeth-TweeSteden Hospital (ETZ) at Tilburg, The Netherlands. The patients underwent Gamma Knife Radiosurgery (GKRS) at the Gamma Knife Center. This study was approved by the ETZ science office and by the Ethics Review Board at Tilburg University. We aimed to look for data on all the factors identified from the literature for the SRT group. Out of these factors, we collected the data for the variables for which data was available for analysis at ETZ. The patients for whom there was incomplete data for this subset of variables were excluded from the data set. The data were randomly split into training and testing data sets. For the treatment dose, we took the average value from the dose range. Similarly, we took the mean tumor volume across the metastases for patients with more than one brain metastasis. The data was normalized and supplied to the Random Forest classifier. The model was trained with the training data set and then tested with the test data set.

The performance of the model was evaluated by measuring the following metrics: classification accuracy, precision, recall, and Area Under the Receiver Operating Characteristic (ROC) Curve (AUC). The ratio of the number of correct predictions to the total number of input samples determines the accuracy of a machine learning algorithm. The precision is the ratio of true positive predictions to the total number of positive predictions made by the model, while recall is the ratio of true positive predictions to the total number of actual positives in the dataset. ROC is a graphical plot created by plotting the true positive rate vs. the false positive rate at various threshold settings. AUC refers to the area under the ROC curve.

K-fold cross validation was applied to the model. It provides a robust estimate of a model’s performance by partitioning the dataset into k subsets (folds) and iteratively training and evaluating the model on different combinations of training and validation sets. Cross-validation helps in obtaining a more reliable performance metric than a single train-test split. The different values used for K were 3, 5, and 10. The average accuracy, precision, recall and AUC across the different folds was calculated. We also extracted the importance of the various factors for predicting the LC from the trained model.

## Results

### Selected studies

**Fig. 1:**
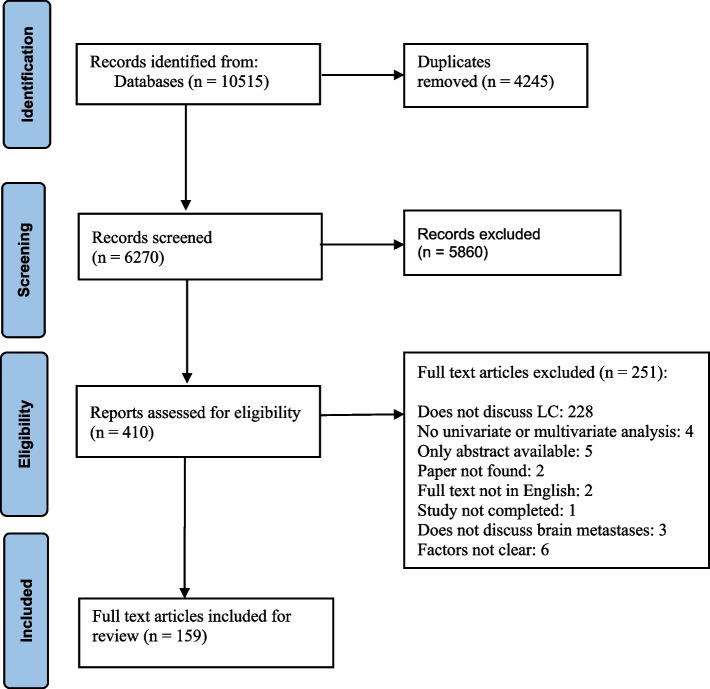
PRISMA flowchart of the study selection

The systematic literature search identified 6270 unique records (Fig. 1). After screening the title and abstract, 410 full texts were considered, and ultimately 159 studies were included in the review (Fig. 1).

### Study characteristics

**Fig. 2:**
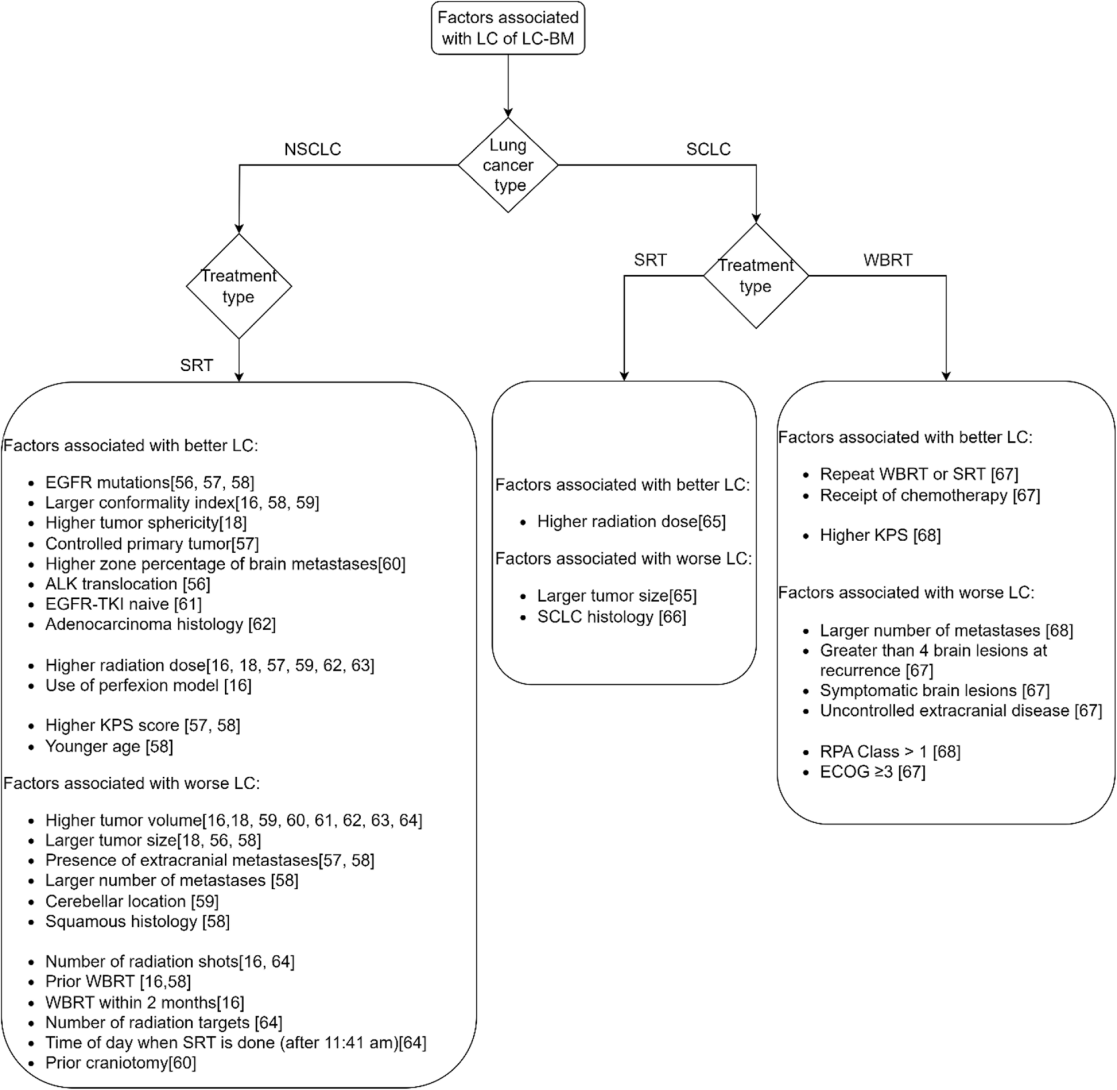
The factors associated with LC of Lung cancer brain metastases

**Fig. 3:**
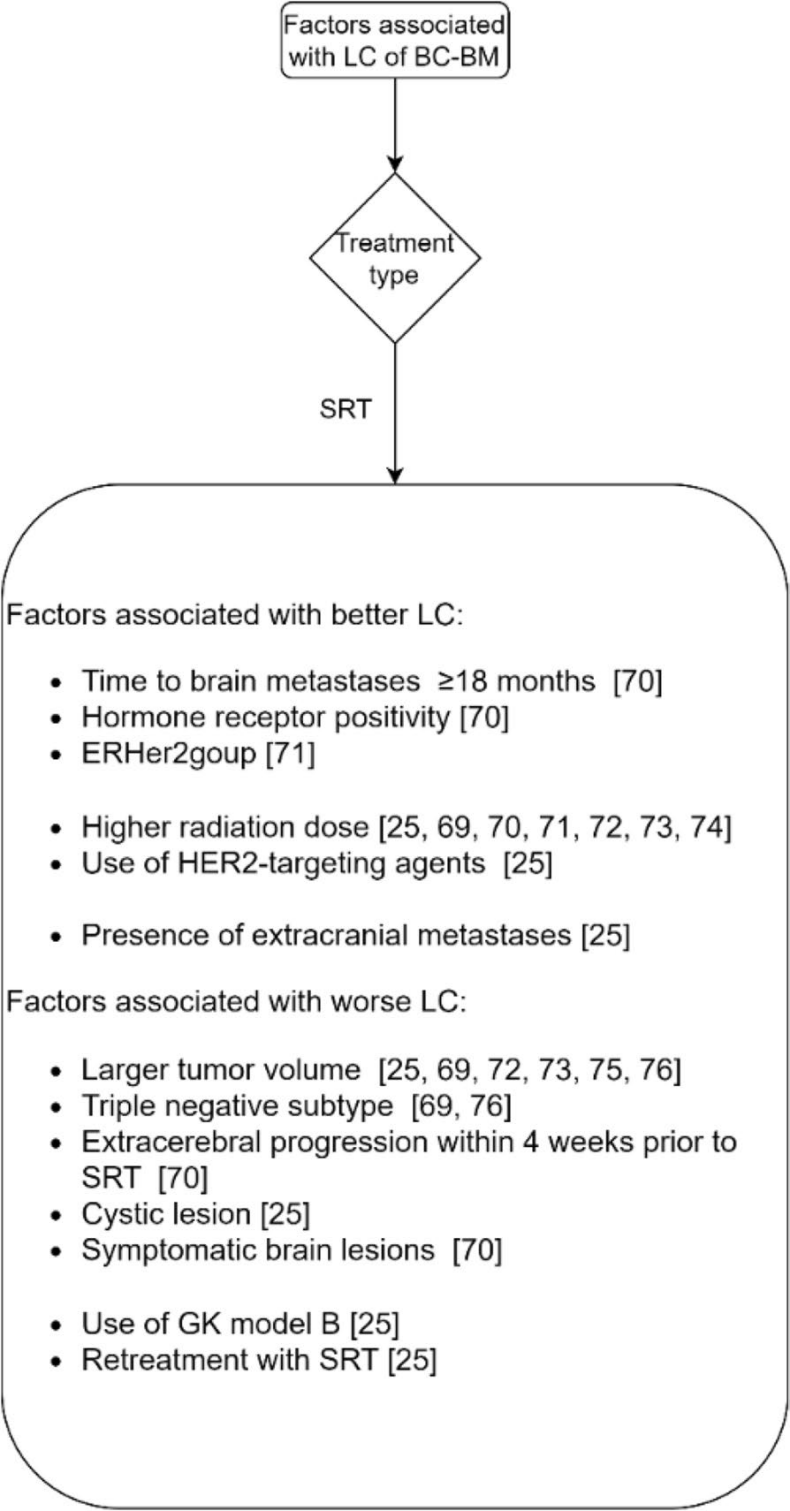
The factors associated with LC of breast cancer brain metastases

**Fig. 4:**
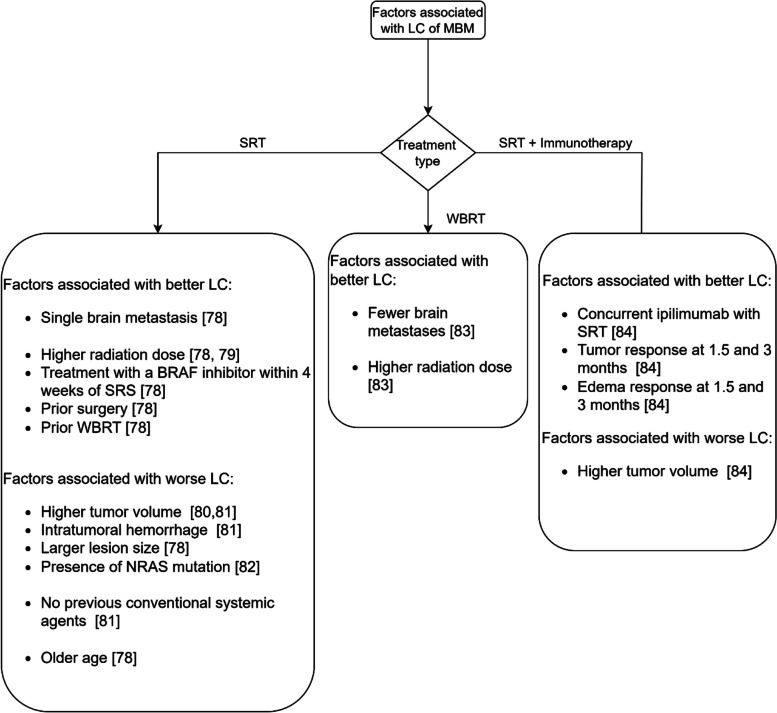
The factors associated with LC of melanoma brain metastases

**Fig. 5:**
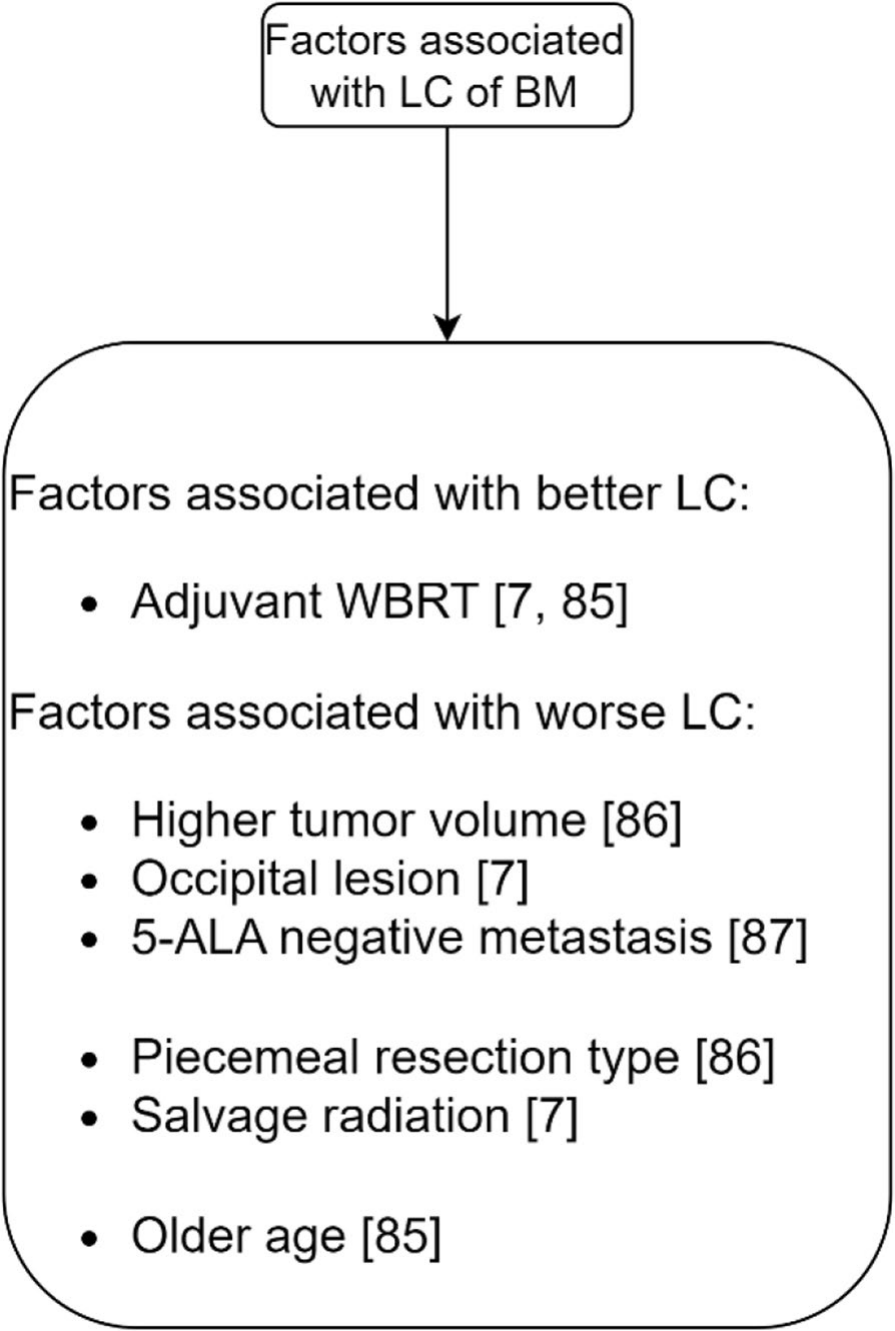
The factors associated with LC after surgery

**Fig. 6:**
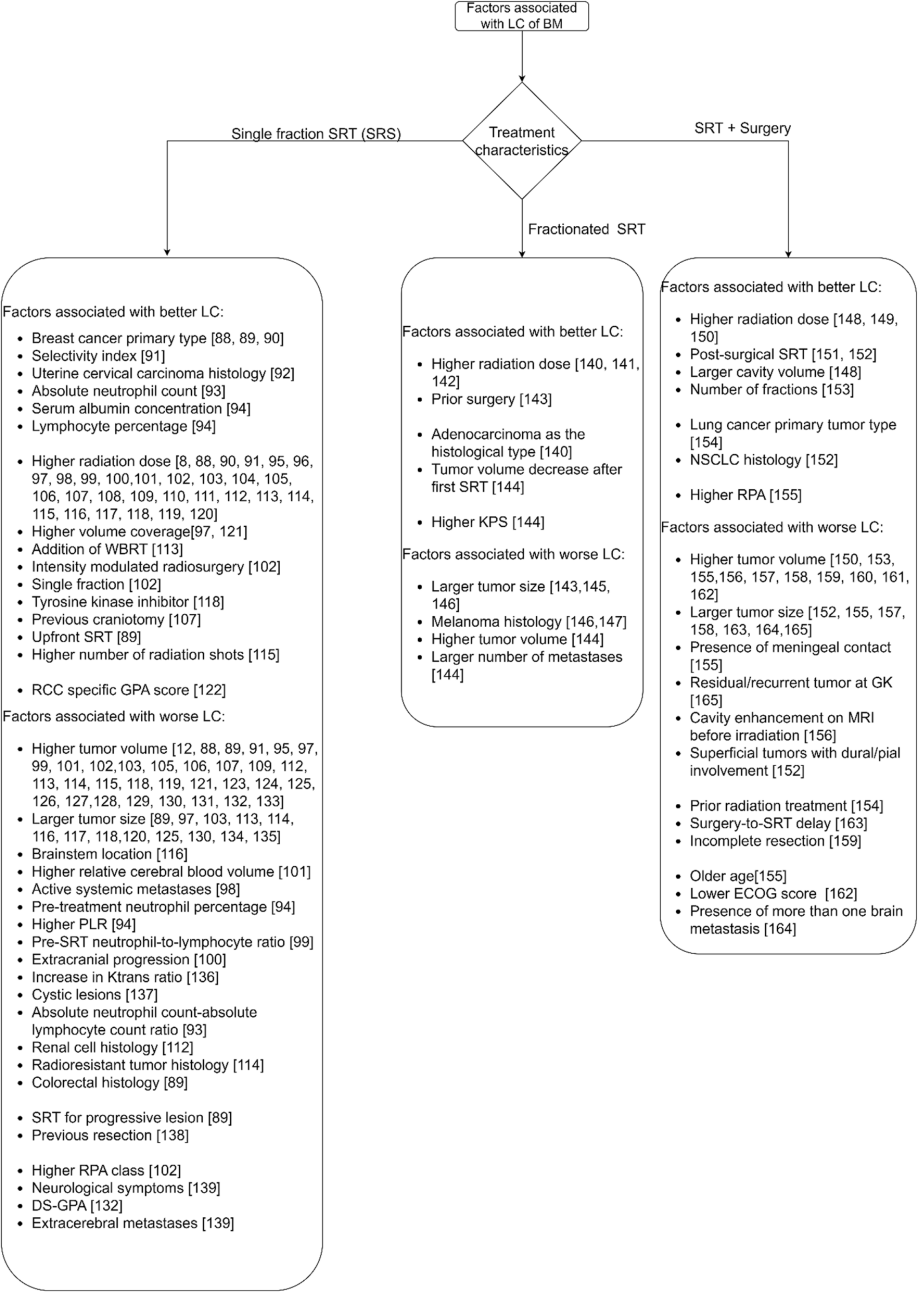
The factors associated with LC after SRT

**Fig. 7:**
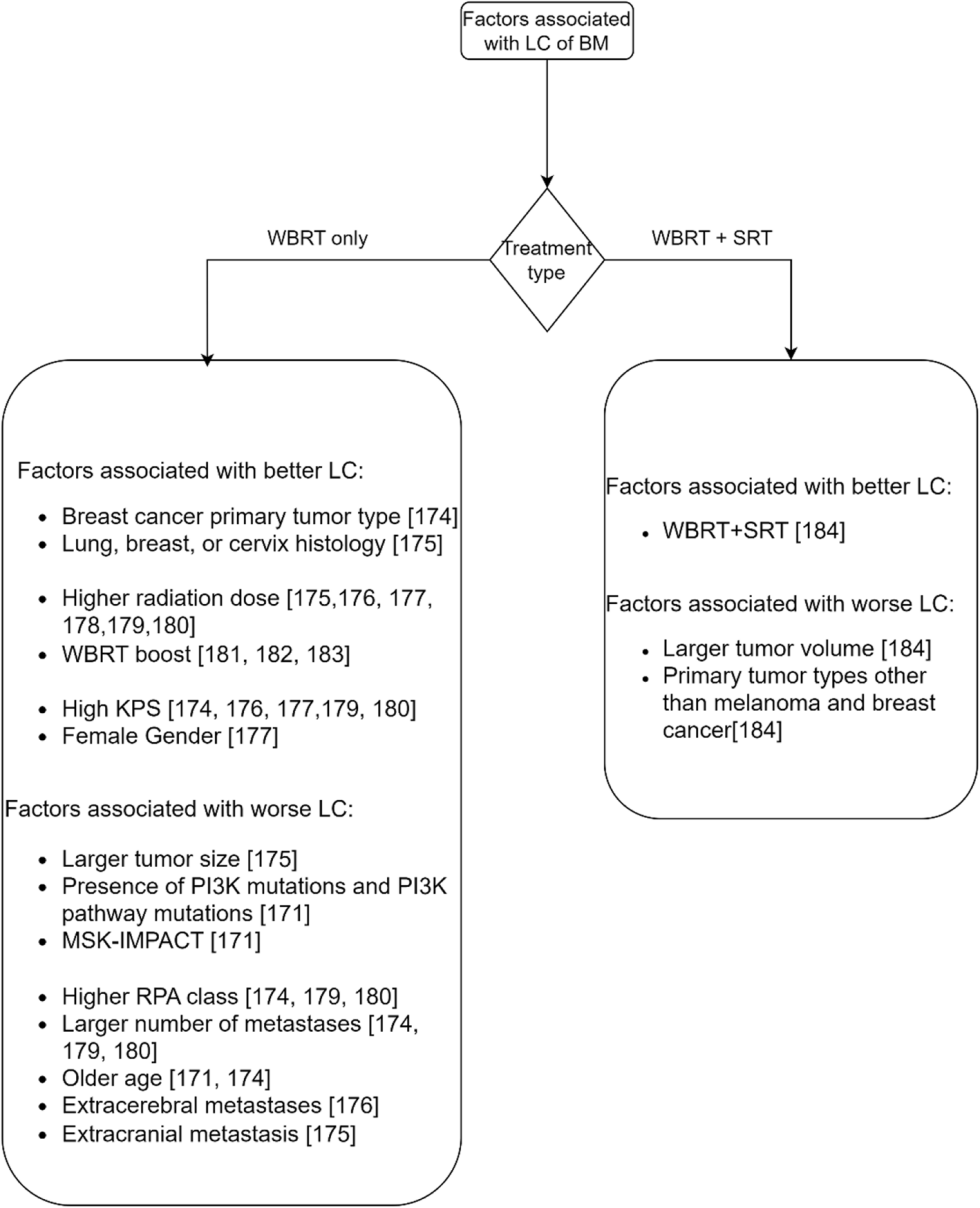
The factors associated with LC after WBRT

The included 159 studies were heterogeneous, covering a wide range of primary cancer types and were distributed across the different treatment types available for brain metastases. The three groups that we created based on the primary tumor types were melanoma, breast cancer, and lung cancer. The significant factors associated with LC in these groups are presented as flowcharts in Figs. 2, 3, and 4. The different groups that we created based on the treatment types were a surgery, an SRT, and a WBRT group. The significant factors associated with LC in these groups are visually depicted in Figs. 5, 6, and 7. Within these figures, the factors are aggregated based on their characteristic type. The findings per group are discussed below. The studies which did not find any significant factors [31–43] were not included in these groups. Also, comparative studies that did not find any significant factors other than the treatment type [44–54] were not included in these groups and are not further discussed.

### Primary tumor histology

#### Lung cancer brain metastases

Lung cancer is the leading cause of cancer-related death [16]. In addition, lung cancer is the most common malignancy giving rise to brain metastases, accounting for 40 to 60% of all cases of brain metastases [55]. Fig. 2 summarizes the factors associated with the LC of brain metastases from lung cancer. We distinguished the factors for Non-Small Cell Lung Cancer (NSCLC) from those for Small Cell Lung Cancer (SCLC).

##### NSCLC

The factors associated with LC for brain metastases from NSCLC after treatment with SRT are included in Fig. 2. There are no papers that discuss the LC factors after treatment with WBRT. The factors that were not associated with LC for NSCLC brain metastases after SRT are gender [16, 18, 57, 59, 60], chemotherapy [57, 60], Graded Prognostic Assessment (GPA) score [16, 18], and Recursive Partitioning Analysis (RPA) class [16, 18].

There are also factors for which there are contrasting findings: some studies found them to be associated with LC, whereas others showed that they are not associated with LC. In contrast with the studies reported in Fig. 2, other studies did not find an association with LC for location [16, 18, 60, 83], extracranial metastases [18], Karnofsky Performance Scale (KPS) score [18, 60], prior WBRT [57, 59], tumor volume [57], and prior craniotomy [57].

##### SCLC

Figure 2 also includes the factors that are associated with LC for brain metastases from SCLC after treatment with SRT or WBRT. In contrast with the study [67] reported in Fig. 2 that suggested that uncontrolled extracranial metastases are associated with worse LC, another study [68] suggested that presence of extracranial metastases are not associated with LC.

#### Breast cancer brain metastases

Breast Cancer (BC) is the second most common cause of brain metastases in approximately 30% of all women with brain metastases [69]. The incidence of brain metastases appears to be increasing, likely due to earlier diagnosis and prolonged survival with contemporary treatments of BC [69]. Fig. 3 summarizes the factors associated with the LC of brain metastases from BC.

For treatment with SRT, the factors that are not associated with LC are prior WBRT [25, 70, 71, 76], surgery prior to SRT [25, 70, 76], and age [25, 70, 73]. There are mixed findings about the association of Her2 positivity with the LC of brain metastases from BC [70, 71, 74]. There are also mixed findings about the association of the number of metastases with LC [25, 69].

In contrast with the studies reported in Fig. 3, one other study did not find an association with LC for radiation dose [76].

Only one study examined LC for BC brain metastases treated with WBRT. This study reported that a higher KPS score, and higher RPA class are associated with better LC.

#### Melanoma brain metastases

Thirty-four percent of patients with melanoma developed brain metastases in clinical studies [77]. With a median overall survival of 4.6 months, brain metastases are the leading cause of death in melanoma patients [77]. In addition, the management of melanoma brain metastases remains challenging because of its resistance to radiotherapy and chemotherapies [77].

The factors associated with the LC of melanoma brain metastases are shown in Fig. 4. Gender [81], age [81], location of brain metastases [81], and WBRT [79, 80] do not seem to be associated with LC of melanoma brain metastases after treatment with SRT.

In contrast with the studies reported in Fig. 4, other studies did not find an association with LC for tumor volume [79], tumor size [79], and SRT dose [80, 81] for treatment with SRT. There are mixed findings about the association of BRAF mutation with LC [78, 79, 82].

### Treatment type

#### Surgery

Surgery is a treatment option for large metastatic brain lesions [7]. Fig. 5 summarizes the factors associated with LC after surgery.

The factors that are not associated with LC after surgical resection are gender [86], and KPS score [86].

#### Stereotactic radiotherapy

Figure 6 summarizes the factors associated with LC after treatment with SRT. The term SRT is used for both single fraction (also called Stereotactic Radiosurgery (SRS)) and fractionated stereotactic radiotherapy. Single fraction SRT (SRS) is a specialized radiation therapy that delivers a single, high dose of radiation directly to the tumor. Fractionated stereotactic radiotherapy delivers multiple, smaller doses of radiation over time. The studies on the factors associated with LC after SRT are sub-grouped into three categories namely: single fraction SRT (SRS), fractionated SRT, and SRT with surgery.

##### Single fraction SRT(SRS)

The factors that are associated with better LC after SRS are shown in Fig. 6, along with the factors that are associated with worse LC after SRS. The factors that are not associated with LC are chemotherapy [97, 98, 100, 126, 166], primary tumor status [100, 104, 124], GPA [127, 167], systemic treatment [95, 99, 106], time interval from primary tumor diagnosis to brain metastases [100, 110], use of targeted agents [106], energy index [8], DS-GPA [95, 110], and laterality [121, 167].

In contrast with the studies reported in Fig. 6, other studies did not find an association with LC after single fraction SRT (SRS) for dose [89, 102, 124, 125, 127, 166–168], tumor location [97, 98, 105–107, 109, 113, 121, 125, 129, 167], KPS [95, 96, 99, 101, 104, 110, 124, 126, 166, 167], primary tumor type [95–97, 99, 101, 107, 109, 111, 125, 127, 167, 168], tumor volume [8, 98, 100, 102, 104, 112, 128, 166, 167, 169], extracranial metastases [95–97, 99, 100, 166, 167], tumor size [95, 101, 168], WBRT [97, 100, 103, 104, 124], RPA class [95, 96, 100, 167], primary tumor location [105, 110, 117], breast cancer primary tumor type [103], conformity index [102, 119], number of fractions [100], presence of systemic metastases [110], and NSCLC primary tumor type [89].

There are mixed findings about the association of the number of metastases [95–98, 100, 103, 110, 112, 135, 166, 167], Paddick’s conformality index [113, 139, 169], prior WBRT [98, 99, 102, 107, 110, 111, 117, 121, 126, 166, 170], lung cancer primary tumor type [102, 103, 108] and age [89, 90, 95–99, 101–107, 110, 121, 124, 126, 127, 135, 166, 167, 170] with LC. There are also contrasting findings about the association of gender [95, 96, 98–103, 105, 106, 127, 133, 138, 166, 167] and melanoma histology [89, 98, 102, 103, 112, 120, 121, 126, 138] with LC.

##### Fractionated SRT

The factors that are associated with better LC after treatment with fractionated SRT are shown in Fig. 6. The figure also includes the factors that are associated with worse LC. The factor that is not associated with LC is systemic treatment [140].

There are mixed findings about the association of primary tumor histology with the LC of brain metastases [140, 146]. There are also mixed findings about the association of number of fractions with the LC of brain metastases [147, 171]. In contrast with the studies reported in Fig. 6, other studies did not find an association with LC for dose [158, 172].

##### SRT with surgery

Figure 6 also includes the factors that are associated with better and worse LC after treatment with SRT after surgery.

The factors that are not associated with LC are gender [150, 160], piecemeal excision [156], radioresistant primary tumor type [160, 163], and the time interval between surgery and SRS [152, 160].

There are mixed findings about the association of GPA score [155, 156] and the margin around the resection cavity [157, 173] with the LC of brain metastases.

In contrast with the studies reported in Fig. 6, other studies did not find a significant association between LC and tumor location [152, 160, 165], histology [150, 156, 165], age [150, 160], residual tumor [150], GPA [150], dose [165], number of metastases [160], tumor volume [165], and tumor size [165].

#### Whole brain radiation therapy

Figure 7 shows the factors associated with LC of brain metastases after treatment with WBRT. The WBRT treatment group is classified into two subgroups, namely: treatment with WBRT alone, and combination of WBRT with SRT.

In contrast with the studies reported in Fig. 7, other studies did not find an association with LC for age [175–180, 185], gender [174–176, 178–180], extracerebral metastases [174, 177, 179, 180, 185], extracranial metastasis [178], number of metastases [177, 178, 185], the interval from first diagnosis to WBRT [174, 177, 179], KPS [178, 185], primary tumor type [177, 179] and RPA class [185] for treatment with WBRT alone.

Combining WBRT with SRS was found to be associated with better LC when compared to treatment with WBRT alone. For this treatment combination, the factors not associated with LC are age [184], and gender [184].

### Summary of results

Higher SRT radiation dose was found to be associated with better LC in lung cancer, breast cancer, and melanoma primary tumor groups. Also, in the SRT group (in which multiple primary tumor types were included), a higher dose was reported to be associated with better LC. Although few studies did not find any association of SRT radiation dose, many studies in the literature suggest that a higher SRT radiation dose is associated with better LC.

Table 1 summarizes the factors for which there is univocal evidence from the literature (meaning there is neither mixed nor contrasting findings) to suggest their association with better LC.

**Table 1:** Factors that are associated with better LC

Primary tumor/treatment group	Factors
Lung cancer (NSCLC)	Higher SRT radiation dose, larger conformality index, presence of EGFR mutations
Lung cancer (SCLC)	Higher SRT radiation dose
Breast cancer	Higher SRT radiation dose
Melanoma	Higher SRT radiation dose
WBRT	Higher KPS score, breast cancer primary tumor type, WBRT boost
SRT	Higher radiation dose, higher KPS score
Surgery	Adjuvant WBRT

On the other hand, higher tumor volume seems to be associated with worse LC in the SRT group. Table 2 summarizes the factors for which there is univocal evidence from the literature to suggest their association with worse LC.

**Table 2:** Factors that are associated with worse LC

Primary tumor/treatment group	Factors
Lung cancer (NSCLC)	Larger tumor size, number of radiation shots
Breast cancer	Presence of triple-negative breast cancer subtype
Melanoma	Presence of intratumoral hemorrhage
WBRT	Higher RPA class
SRT	Higher tumor volume, larger tumor size, recurrent lesion

This review showed that some of the significant factors in some studies are found to be non-significant in others. The factors for which there is such mixed evidence of their association with LC are a higher number of brain metastases for the breast cancer group, the presence of extracranial metastases and prior WBRT for the lung cancer group, a larger tumor size for the melanoma group, gender, and number of metastases for WBRT treatment group and prior WBRT, multifraction and number of metastases for SRT group.

Some factors are significant in some studies but found to be non-significant in a higher number of other studies. These factors for which there is only such weak evidence of their association with LC are female gender, older age for the WBRT treatment group, and breast cancer primary type, NSCLC primary tumor histology, a higher number of metastases, melanoma histology, and older age for the SRT group.

### Machine learning use case

**Fig. 8:**
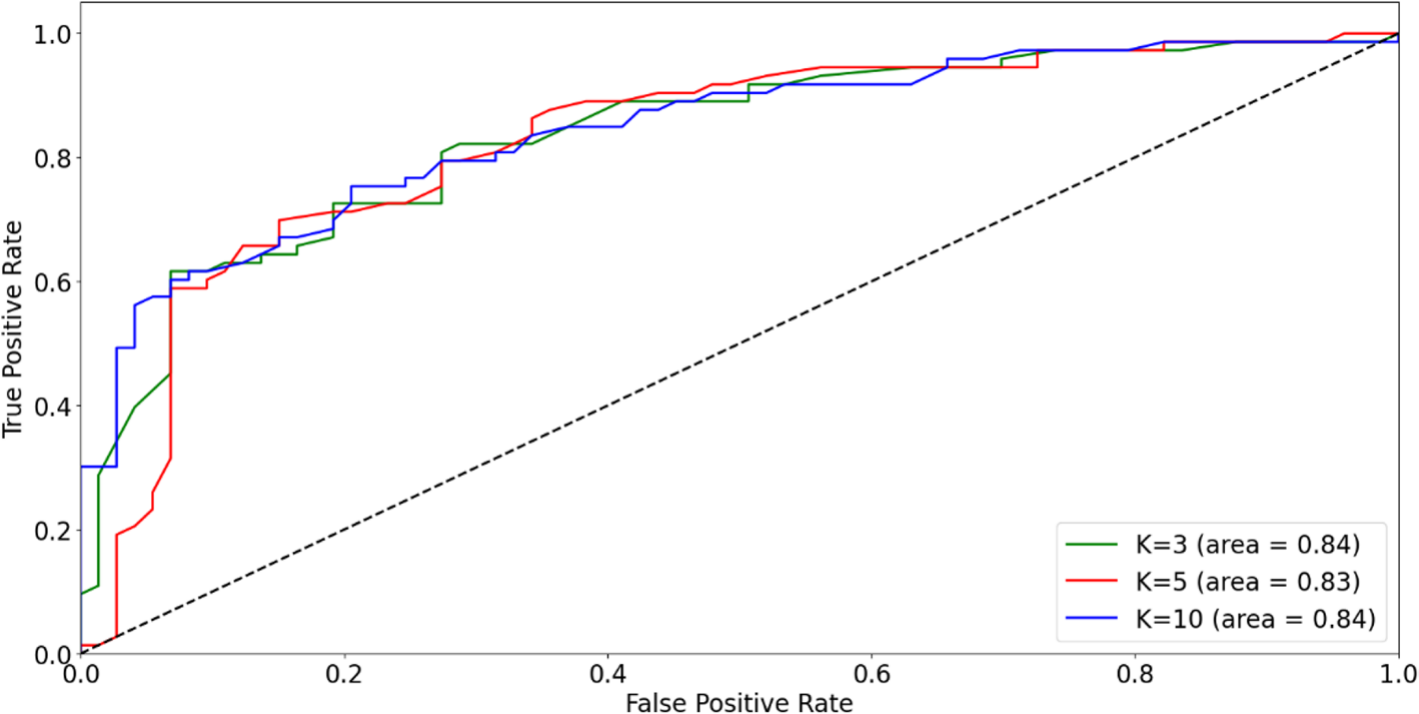
ROC curve for the prediction model

**Fig. 9:**
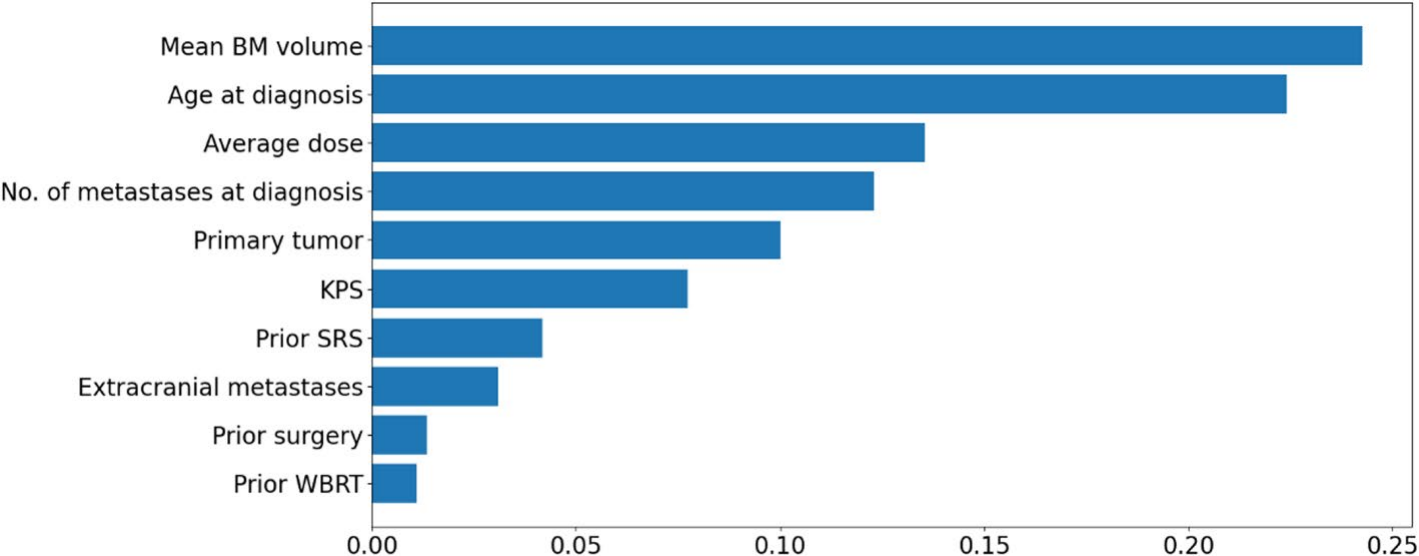
Variable importance for LC in decreasing order of significance

The list of all factors associated with LC after SRT for brain metastases from a diversity of primary cancers is illustrated in Fig. 6. Out of these factors, the variables for which data was available for analysis at ETZ were age, KPS score, number of brain metastases, average brain metastases volume, primary tumor type, presence of extracranial metastases, average treatment dose, prior WBRT, prior surgery, and prior SRS. The patients with incomplete data for this subset of variables were excluded from the data set. After this filtering, we had 135 patients with complete data. Table 3 summarizes the characteristics of these patients. The patient cohort was randomly partitioned into training and testing datasets, comprising 121 and 14 patients, respectively. A Random Forest model was trained iteratively on the training dataset and evaluated on distinct validation sets within the training data. The assessment of its classification accuracy revealed an average accuracy of 80% across the diverse folds utilized in the cross-validation procedure. The average precision and recall across the folds were 75% and 81.6% respectively. The AUC across the folds is depicted in Fig. 8. The average AUC across the three folds was 0.84. The most important factor as per the algorithm was the tumor volume followed by age and average SRT dose. The presence of prior WBRT was the least significant factor as per the algorithm (Fig. 9).

**Table 3:** Patient characteristics

**Age (years)**	
Average	63
Minimum	39
Maximum	85
**Sex**	
Male	58
Female	77
**KPS**	
60	3
70	17
80	32
90	42
100	41
**Number of tumors**	
1	33
2-3	51
4-10	44
>10	7
**Primary cancer**	
Lung	89
Melanoma	7
Breast	5
Others	34
**Presence of extracranial metastases**	
Yes	61
No	74
**Prior SRS**	
Yes	15
No	120
**Prior surgery**	
Yes	9
No	126
**Prior WBRT**	
Yes	8
No	127
**Tumor volume(mm3)**	
Average	16752
Minimum	88
Maximum	88029
**Average SRS dose(Gy)**	
Average	22.018
Minimum	14
Maximum	25

## Discussion

The aim of this study was to conduct a comprehensive review of factors associated with LC of brain metastases, categorizing them across various primary tumor types and treatment modalities. By systematically analyzing a wide array of literature, we aimed to identify and present factors associated with LC, offering a holistic perspective. Additionally, we explored the translational potential of this knowledge in the context of machine learning, demonstrating its practical utility by applying insights to the stereotactic radiotherapy group.

One hundred and fifty-nine studies were included in the review. All the factors associated with LC of brain metastases were explored without restrictions on the primary tumor types, treatment types and study methodology. Also, we reviewed all the characteristics associated with LC and did not limit them to one type of characteristics, for instance treatment, patient, brain metastases or primary tumor characteristics. The studies were grouped based on primary tumor type. Studies that did not focus on a specific primary tumor type or included heterogeneous groups of patients with different primary cancers were grouped based on treatment type.

The results showed numerous significant factors for each group, underscoring the complexity of LC determinants. Notably, some factors showed significance in certain studies but not in others, highlighting the need for further investigation into factors contributing to these discrepancies, including patient, tumor, and treatment characteristics and their potential interactions. Additionally, differing international guidelines may have contributed to variations in study outcomes. The data from older studies show that a wide variety of SRT doses was used in the past [194, 195]. However, recently, national and international guidelines were developed to increase the homogeneity of the treatment [188, 189, 193]. Despite these recent guidelines, there are still minor differences between dosage guidelines across the countries. For instance, for a tumor with a volume of 20 cm3, the dosage guideline in US and UK is 15 Gy, while in Netherlands it is 18 Gy [188, 189, 193].

To illustrate the practical utility of our findings, we used the factors generally associated with LC for feature selection in a Random Forest machine learning algorithm for the SRT group. The list of factors identified in this review served as an input for us to extract the features for the algorithm. The resulting Random Forest model predicted the LC of brain metastases with an accuracy of 80% and an AUC of 0.84. The neural network model proposed by Kawahara et al. [19] for predicting the local response of metastatic brain tumors to SRT, built with 45 patient samples, provided a prediction accuracy of 78% for the evaluation dataset. The machine learning model trained with the clinical features of 100 patients in the study of Jaberipour et al. [24] reached a prediction accuracy of 63%. The prediction model developed by Jalalifar et al. [187] with clinical features of 99 patients had a prediction accuracy of 67.5%. The higher prediction accuracy of the Random Forest algorithm trained with the features selected from the systematic review utilized in this study suggests that our feature selection could help to increase the prediction accuracy of the machine learning algorithms. This prediction model holds promise for clinicians, offering a valuable tool to predict personalized treatment outcomes and to foresee the impact of changes in treatment characteristics such as dose, and prior brain treatments. As per the algorithm, the most important factor was tumor volume, while presence of prior WBRT was the least important factor.

Understanding the factors associated with LC is crucial, given its link to improved overall survival [186]. Our study advocates extending this approach for the SRT group to the other treatment and primary tumor groups described in this review. This could be the scope of a future study on this topic. Knowing the factors associated with the LC of brain metastases is imperative to predict the overall survival of the patients and in some cases to prolong survival if the factors are controllable. Moreover, the streamlined approach of using the identified factors has the potential to significantly facilitate and enhance efficiency in future machine learning studies, reducing time and computational costs during the data extraction and feature selection process. Limiting the number of features could also improve the interpretability of machine learning algorithms.

A limitation of this study is that we included only the clinical features for training the machine learning algorithm. The addition of imaging features from the pre-treatment MRI scans could increase the prediction performance of the machine learning algorithm. Also, for a more rigorous evaluation of the efficacy and robustness of the models, further investigations should be performed on larger patient cohorts, preferably with multi-institutional data. An external validation data set could also improve the generalizability of the prediction model.

In conclusion, our study expands clinical insights into LC in brain metastases and bridges a gap between traditional clinical understanding and advanced machine learning applications. The identified factors are a foundation for future advancements in predictive modeling and treatment optimization, fostering a more personalized and effective approach to cancer care.

### Supplementary Information

Supplementary Material 1.

## Data Availability

The data used for this study is available at ETZ and is accessible after approval from the ETZ Science office.

## Abbreviations

LC: Local Control
BM: Brain Metastases
WBRT: Whole brain radiotherapy
SRT: Stereotactic radiotherapy
DC: Distant Control
OS: Overall Survival
PFS: Progression-Free Survival
iPFS: Intracranial Progression-Free Survival
NSCLC: Non-Small Cell Lung Cancer
SCLC: Small Cell Lung Cancer
ECOG: Eastern Cooperative Oncology Group
RPA class: Recursive Partitioning Analysis
KPS score: Karnofsky Performance Scale
GPA score: Graded Prognostic Assessment
BC: Breast Cancer
GK: Gamma Knife
SRS: Stereotactic Radiosurgery
GTV: Gross Tumor Volume
PLR: Platelet-to-Lymphocyte Ratio
ANC/ALC/NLR: Absolute Neutrophil Count/Absolute Lymphocyte Count/ratio
TT/IT: Targeted or immunotherapy
MRI: Magnetic resonance imaging
EANO: European Association of Neuro-Oncology
ESMO: European Society for Medical Oncology
ROC: Receiver Operating Characteristic
AUC: Area Under the Receiver Operating Characteristic Curve
