# Supplementary material for: Factors associated with the local control of brain metastases: a systematic search and machine learning application
Source: BMC Med Inform Decis Mak. 2024 Jun 21;24:177. doi: 10.1186/s12911-024-02579-z (PMC11191176; doi:10.1186/s12911-024-02579-z)
Supplement: Supplementary file 1 — Supplementary Material 1. [file 12911_2024_2579_MOESM1_ESM.pdf]

SUPPLEMENTARY TABLES

Supplementary Table 1. Inclusion and exclusion criteria in terms of PICOs
----

**Patients**	All patients with brain metastases<br><br>Any primary tumor<br><br>No restriction for prognosis, country, or clinical setting
**Intervention**	Prognostic factors
**Comparison**	Impact on local control
**Outcomes**	Local control (e.g.: recurrence, growth)

PICO: patient, intervention, comparison, outcome.

Supplementary Table 2. Search strategies

**Database**	**Search**
PubMed	(brain neoplasms/secondary[MeSH Terms] OR Infratentorial Neoplasms /secondary[MeSH Terms] OR Brain metasta*[Tiab] OR Brain metastas*[Tiab] OR cerebral metasta*[Tiab] OR Intracranial metasta*[Tiab])<br><br>AND<br><br>(risk factors[Tiab] OR prognostic[Tiab] OR prognosis[Tiab] OR markers[Tiab] OR predict*[Tiab]) AND<br><br>(recurrence[Tiab] OR regrowth[Tiab] OR growth[Tiab] OR control[Tiab] OR progression[Tiab] OR outcome[Tiab] OR follow-up[Tiab] OR progressive disease[Tiab])
Cochrane	#1 MeSH descriptor: [Brain Neoplasms] explode all trees and with qualifier(s): [secondary - SC]<br><br>#2 MeSH descriptor: [Infratentorial Neoplasms] explode all trees and with qualifier(s): [secondary - SC]<br><br>#3 ((Brain metasta* OR Brain metastas* OR cerebral metasta* OR Intracranial metasta*):ti,ab)<br><br>#4 ((risk factors OR prognostic OR prognosis OR markers OR predict*):ti,ab)

	#5 ((recurrence OR regrowth OR growth OR control OR progression OR outcome OR follow-up OR progressive disease):ti,ab)<br><br>#6 #1 OR #2 OR #3 AND #4 AND #5
Embase	# 1 (('brain metastasis'/exp OR ‘Brain metasta*’ OR ‘Brain metastas*’ OR ‘cerebral metasta*’ OR ‘Intracranial metasta*’):ab,ti) AND<br><br># 2 ((‘risk factors’ OR prognostic OR prognosis OR markers OR predict*):ab,ti) AND<br><br>#3 ((recurrence OR regrowth OR growth OR control OR progression OR outcome OR ‘follow up’ OR ‘progressive disease’):ab,ti)<br><br>#1 AND #2 AND #3 AND [humans]/lim AND [english]/lim AND ([article]/lim OR [data papers]/lim OR [review]/lim) AND [abstracts]/lim
Web of science	#1 (((((TI=("brain neoplasms/secondary"[MeSH] OR “Infratentorial Neoplasms /secondary[MeSH Terms]” OR Brain metasta* OR Brain metastas* OR cerebral metasta* OR Intracranial metasta*) AND<br><br>TI=(risk factors OR prognostic OR prognosis OR markers OR predict*) AND<br><br>TI=(recurrence OR regrowth OR growth OR control OR progression OR outcome OR follow-up OR progressive disease)))))) AND LANGUAGE: (English) AND DOCUMENT TYPES: (Article OR Early Access OR Review) Indexes=SCI-EXPANDED, SSCI, A&HCI, BKCI-S, BKCI-SSH, ESCI Timespan=All years<br><br>#2 (((((AB=("brain neoplasms/secondary"[MeSH] OR “Infratentorial Neoplasms /secondary[MeSH Terms]” OR Brain metasta* OR Brain metastas* OR cerebral metasta* OR Intracranial metasta*) AND<br><br>AB=(risk factors OR prognostic OR prognosis OR markers OR predict*) AND<br><br>AB=(recurrence OR regrowth OR growth OR control OR progression OR outcome OR follow-up OR progressive disease)))))) AND LANGUAGE: (English) AND DOCUMENT TYPES: (Article OR Early Access OR Review) Indexes=SCI-EXPANDED, SSCI, A&HCI, BKCI-S, BKCI-SSH, ESCI Timespan=All years<br><br>#3 (#1 OR #2)
